# Epigenetic analysis in rheumatoid arthritis synoviocytes

**DOI:** 10.1038/s12276-019-0215-5

**Published:** 2019-02-22

**Authors:** Seokjin Ham, Jae-Bum Bae, Suman Lee, Bong-Jo Kim, Bok-Ghee Han, Seung-Ki Kwok, Tae-Young Roh

**Affiliations:** 1 Department of Life Sciences, POSTECH, Pohang, 37674 Korea; 2 Division of Genome Research, Center for Genome Science, Korea National Institute of Health, Korea Centers for Disease Control and Prevention, Osong, 28160 Korea; 3 Division of Rheumatology, Department of Internal Medicine, Seoul St. Mary’s Hospital, College of Medicine, The Catholic University of Korea, Seoul, 06591 Korea; 4 Division of Integrative Biosciences and Biotechnology, POSTECH, Pohang, 37674 Korea

**Keywords:** Rheumatoid arthritis, Epigenetics

## Abstract

Rheumatoid arthritis (RA) is a complex chronic systematic disease with progressive destruction of the joints by invasive synoviocytes. To characterize the key regulators involved in the development of RA, we obtained multilayer epigenomics data including DNA methylation by whole-genome bisulfite sequencing, miRNA profiles, genetic variations by whole-exome sequencing, and mRNA profiles from synoviocytes of RA and osteoarthritis (OA) patients. The overall DNA methylation patterns were not much different between RA and OA, but 523 low-methylated regions (LMRs) were specific to RA. The LMRs were preferentially localized at the 5′ introns and overlapped with transcription factor binding motifs for GLI1, RUNX2, and TFAP2A/C. Single base-scale differentially methylated CpGs were linked with several networks related to wound response, tissue development, collagen fibril organization, and the TGF-β receptor signaling pathway. Further, the DNA methylation of 201 CpGs was significantly correlated with 27 expressed miRNA genes. Our interpretation of epigenomic data of the synoviocytes from RA and OA patients is an informative resource to further investigate regulatory elements and biomarkers responsible for the pathophysiology of RA and OA.

## Rheumatoid arthritis: Understanding the regulation of disease-relevant genes

Whole genome analysis of synoviocytes, specialized cells in the joint-lubricating synovial fluid, sheds light on the pathogenic mechanisms of rheumatoid arthritis (RA). Around 350 million people worldwide suffer joint pain and stiffness due to RA, but the inheritance pattern of the disease remains unclear. A study led by Tae-Young Roh at Pohang University of Science and Technology, South Korea, reveals a distinct pattern of chemical tags on the DNA of synoviocytes from RA patients. Differences in methyl group tags in over 500 regions of the genome influenced the expression of RA-associated genes and of microRNAs, small RNA molecules that are also involved in the regulation of gene expression. These differentially methylated sites may not only represent potential disease biomarkers, but also offer new insights into the regulation of RA-relevant genes.

## Introduction

Rheumatoid arthritis (RA) is characterized by a progressive destruction of joints by invasive synoviocytes. It is not only a chronic systematic disease but also a complex genetic disease. Approximately 1% of the world population currently suffers from this disease. Genome-wide association studies have identified meaningful genetic variants relevant to RA^1–3^. Mutations in the major histocompatibility complex, class II gene *HLA-DRB1*, are common signatures for the greatest genetic risk^4^. Nevertheless, genetic discoveries are not sufficient to completely explain the disease. The finding that the concordance rate of RA for monozygotic twins is only 15% implies that the inherited DNA sequences alone may not contribute to the low concordance rate^5^. Moreover, gene expression profiles of RA patients are usually heterogeneous^6^. Generally, the markers identified by comparative analysis of gene expression are not always detected in replication studies^7^, suggesting that analysis with only common genetic variants or gene expression profiles is not sufficient for a comprehensive understanding of RA^6^.

Epigenetic modifications are related to many human diseases. Highly dynamic epigenetic changes are controlled by the complex interplay between environmental cues and chromatin context^8^, and they give rise to diverse phenotypes^9^. Epigenetic homeostasis is vital for cells to maintain normal cellular functions. Any uncontrolled epigenetic perturbations can lead to diseases, especially cancers^10,11^. Furthermore, the precise interpretation of the epigenetic landscape is more informative for classification than the simple identification of differentially expressed genes^12,13^. DNA methylation is one of the major epigenetic mechanisms widely examined for understanding the development and diagnosis of human diseases^14^. Several studies have reported the role of DNA methylation in the pathogenesis of RA^15–17^. MicroRNAs (miRNAs) are another important class of epigenetic regulators that ensure robust animal development and homeostasis by fine-tuning the expression of target genes^18^. For example, the expression changes of miRNAs can also contribute to autoimmunity^9^.

However, there have been critical limitations in the previous studies about DNA methylation. The DNA microarray-based results are only valid for a subset of the genome covered by probe sequences on the chip. To overcome this limitation, the whole-genome bisulfite sequencing (WGBS) method was developed to cover all cytosines in the genome and is becoming an easily accessible technique. WGBS allows for comprehensive genomic coverage, high quantitative accuracy, and remarkable reproducibility^19^.

An extensive analysis of WGBS data enables discovery of various types of DNA methylation domains and sites previously not identified. First, partially methylated domains (PMDs) are large continuous regions with low methylation, up to several Mbp in size^20^. PMDs are linked to repressive chromatin and gene silencing^21,22^. Second, more localized hypomethylated regions ranging in size from 5 to 100 kbp, termed DNA methylation valleys (DMVs), are involved in regulating genes encoding transcription factors (TFs) and developmental regulators^23,24^. Finally, smaller localized low-methylated regions (LMRs) are located in distal regulatory regions and usually associated with TF binding^20,25^. Generally, LMRs are not colocalized with CpG islands, and their DNA methylation is inversely correlated with the activity of distal regulatory regions.

Here, we focused on the DNA methylation profiles of synoviocytes isolated from joint replacement surgery of RA and OA patients and identified that some focal methylated regions could be regulatory regions in RA. Using DNA sequences enriched in the focal methylated regions, the potential TF-binding sites associated with DNA methylation were discovered. Single CpG sites with differential methylation patterns can be regarded as potential biomarkers and provide a starting point to examine the epigenetic regulation of disease-relevant genes. The integration of DNA methylation with miRNA expression and exome sequencing data may deepen our current knowledge of RA and OA.

## Materials and methods

### Isolation and primary culture of synoviocytes

Synoviocytes were isolated by enzymatic digestion of synovial tissue specimens obtained from RA and OA patients subjected to total joint replacement surgery. The tissue samples were minced into 2- to 3-mm pieces and treated for 4 h with 4 mg/ml type I collagenase (Worthington, Freehold, NJ, USA) in Dulbecco's modified Eagle's medium (DMEM) at 37 °C under 5% CO_2_. Dissociated cells were centrifuged at 500 × *g*, resuspended in DMEM supplemented with 10% fetal calf serum (FCS), 2 mM l-glutamine, 100 units/ml penicillin, and 100 ng/ml streptomycin and plated in a 75-cm^2^ flask. Only adherent cells were further cultivated in DMEM supplemented with 10% FCS until the number of primary cells reached at least 3–5 × 10^6^, ensuring sufficient genomic DNA and RNA for the DNA methylation and transcriptome analysis. All patients’ samples were collected under the approval of the institutional review board (IRB) at Seoul St. Mary’s Hospital, College of Medicine, the Catholic University of Korea and in accordance with the Declaration of Helsinki. Written informed consent was obtained from all patients.

### Sequencing Library preparation and data generation

Genomic DNA was isolated from the primary cells and treated with sodium bisulfite for the WGBS analysis on the HiSeq2500 platform (Illumina, USA). Another DNA methylation assay was performed using the Infinium Human Methylation 450K BeadChip Kit (Illumina, USA) following the manufacturer’s instructions. The exome was captured by SureSelect Human All Exon V4 + UTRs (Agilent, USA) and subjected to sequencing. miRNA-seq was performed after adding both 5′ and 3′ RNA adapters to each end of the purified small RNA fragments. Gene expression data were obtained with the Human HT-12 v4 Expression BeadChip Kit (Illumina, USA).

### DNA methylation data analysis

**Fig. 1:**
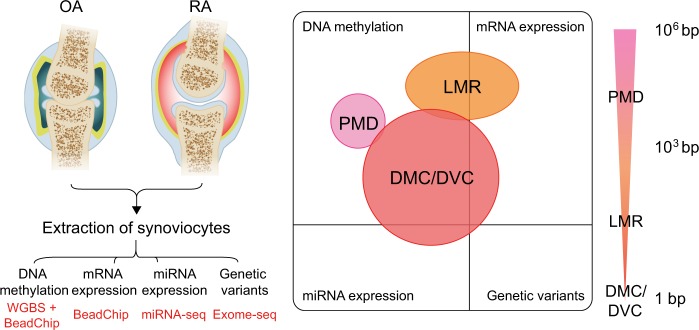
An overview of experimental procedures with RA and OA. The types of data produced in this experiment are DNA methylation, mRNA expression, miRNA expression, and rare genetic variants. Differentially methylated regions (DMR) were separately investigated according to their sizes. The stretching ellipse represents the resolution of each type of DMRs identified. WGBS whole-genome bisulfite sequencing, PMD partially methylated domain, LMR low-methylated region, DMC differentially methylated CpG, DVC differentially variable CpG

The overall experimental procedure is summarized in Fig. 1. The details are provided in the Supplementary Information.

WGBS data were analyzed using the Bismark program (version 0.14.0). The methylation of appropriate internal control markers in the *N4BP2* gene was examined as previously described^26^. Most samples showed typical high methylation except for one sample (OA_120), which was excluded in the following analyses. A hierarchically clustered heatmap was generated with the top 500 CpGs showing the highest variability. Another heatmap was plotted using the recursively partitioned mixture model (RPMM) as previously described^27^.

Broad PMDs for each sample were inspected in a similar manner as previously described^28^. All steps were processed with the MethylSeekR Bioconductor package. PMDs were regarded as shared when a PMD detected in at least one sample of RA colocalized with one found in any OA sample. A PMD detected in only one sample of either RA or OA was defined as specific.

After masking PMDs from the genome, the remaining regions were segmented into smaller methylated regions using MethylSeekR as previously described^28^. The hypomethylated regions were classified into unmethylated regions (UMRs) or locally LMRs according to the number of CpGs they contained (UMRs ≥ 30, LMRs < 30). RA-specific LMRs were denoted as those detected in more than two samples of RA but not in any sample of OA, and vice versa. The levels of methylation around 1.5 kb from the center of specific LMRs were measured, and then, the differential LMRs were dissected by k-means clustering. Genomic annotations of LMRs were performed using the HOMER program (version 4.7.2). The locations of all enhancers inferred from chromatin landscapes^29^ were compared to those of LMRs, and then, the ratio of the overlap in RA-specific LMRs to that in OA-specific LMRs was calculated. The significance was determined by Pearson’s chi-squared test.

To identify LMR-linked sequence features, sequences in the 100 bp flanking regions from the center of specific LMRs were divided into five subsets, which were then used for de novo motif discovery. A heatmap showing motif occurrence in each region was plotted using four subsets except a subset used for the discovery. The relationship between specific LMRs with TF-binding motifs and their target genes was tested by adapting the gene set enrichment analysis (GSEA). With absolute Pearson correlation coefficient >0.5 and permutation *P* < 0.05, direct comparisons between LMRs and genes 5 kb downstream of LMRs were made.

At the nucleotide level, differentially methylated CpGs (DMCs) between RA and OA were identified by logistic regression in the MethylKit with methylation differences >0.2 and *P* < 2.42 × 10^−9^ (0.05/20,605,641, Bonferroni correction). To identify differentially variable CpGs (DVCs) between RA and OA, where the samples in one group showed relatively consistent methylation levels but in the other group had variable methylation levels, Levene’s test in the missMethyl package was performed with FDR < 0.05. The significance of the overlap between DMCs and DVCs was determined by Fisher’s exact test with *P* < 2.2 × 10^−16^ and permutation test with *P* < 0.001.

### Analysis of Infinium Human Methylation 450K BeadChip data

Image data were analyzed using BeadStudio 2.0 (Illumina, USA). Loci on autosomes were retained, but those in any sample with *P* > 0.01 were excluded. Data were processed by color balance adjustment, background level correction, and quantile normalization. After additional filtering steps, the methylation levels in the remaining probes were additionally normalized.

### Network analysis for disease-related pathways

PhenomeExpress was used to build gene expression subnetworks in RA and OA and identify core disease pathways. Disease-relevant phenotypes were used as seeds to construct subnetworks. The phenotypic IDs are as follows: HP:0001370 (rheumatoid arthritis), HP:0002758 (osteoarthritis), MP:0003724 (increased susceptibility to induced arthritis), HP:0002960 (autoimmunity), MP:0001844 (autoimmune response), HP:0008271 (abnormal cartilage collagen), HP:0002829 (arthralgia), HP:0005262 (abnormality of the synovia), MP:0020252 (abnormal collagen level), and ZP:0008630 (abnormally increased rate collagen biosynthetic process). Among the constructed subnetworks, those enriched with specific functions were selected. DMCs and DVCs were overlaid if they were located within genes in the subnetworks.

### miRNA-seq data analysis

miRNA sequencing data were analyzed using miRDeep2 (version 0.0.7). Only adaptor-trimmed reads were aligned to miRBase version 21. Expression of mature miRNAs was normalized to all aligned reads. Differentially expressed miRNAs were identified using the DESeq Bioconductor package with fold change >2 and unadjusted *P* < 0.05. Then, miRNAs expressed in at least 50% of samples were selected. The degree of DNA methylation of miRNAs including 5 kb upstream regions were examined with their expression levels. By applying absolute Spearman correlation coefficient >0.5 and permutation *P* < 0.05, significant miRNA–mRNA pairs were identified. Statistically significant miRNAs were selected by identifying their target mRNAs (negative Spearman correlation coefficient with permutation *P* < 0.05). Using the list of genes targeted by miRNAs, Gene Ontology (GO) analysis was performed by ClueGO (version 2.2.3), and functionally organized subnetworks were constructed with *P* < 0.05. Then, DMCs and DVCs within target genes were determined for further analysis.

### Exome data analysis

Rare genetic variations were detected by the TREVA pipeline^30^. To find RA-specific mutations, only genes with variants found in more than two RA samples were selected. To assess whether a particular variant originated from background noise, Samocha’s statistical framework^31^ for interpretation of de novo mutation was applied with a conservative significance threshold (*P* = 1.0 × 10^−8^).

### Other analysis

The downstream analyses were implemented with R (version 3.1.3; R Foundation for Statistical Computing, Vienna, Austria, www.r-project.org) and Bioconductor packages^32^ version 3.1 (www.bioconductor.org).

## Results

### Global DNA methylation pattern between RA and OA

Thirteen RA and ten OA samples were selected from a large patient cohort in this study (Fig. 1, Supplementary Table S1). For WGBS analysis, 12 RA and 8 OA samples of high quality were selected. The overall sequencing results exhibited 22.2× average coverage and 86.0% of CpGs covered ≥5× on average (Supplementary Table S2). Non-CpG methylation was as low as 0.1%. To assess the bisulfite conversion rate, the methylation of *N4BP2* gene^26^ was measured as an internal control, and samples showing low *N4BP2* methylation levels were excluded, for example, OA_120 (Supplementary Figure S1a).

Individual CpG methylation predominantly displayed the bimodal pattern of most CpGs being fully methylated or unmethylated (Supplementary Figure S1b, c). Methylation profiles from the Infinium 450K array data analysis were highly concordant with those from the WGBS analysis (Supplementary Figure S2). The distribution of average methylation was quite similar between RA and OA. Usually, high methylation variance across samples may contribute to heterogeneity specific to diseases^33^. However, the RA and OA samples showed a similar distribution of standard deviations (SDs) (Supplementary Figure S1d). Furthermore, the unsupervised hierarchical clustering using the top 500 CpGs with the highest methylation variability did not clearly distinguish RA from OA (Supplementary Figure S1e). The result was identical using the RPMM^27^, which is a clustering method specifically developed for DNA methylation (Supplementary Figure S1f). A broad inspection of methylation revealed the presence of PMDs in RA and OA (Supplementary Figure S3). However, very small fractions were specific to each disease. These results indicated that the global methylation pattern is almost similar between RA and OA.

### Focal methylated regions as regulatory regions

**Fig. 2:**
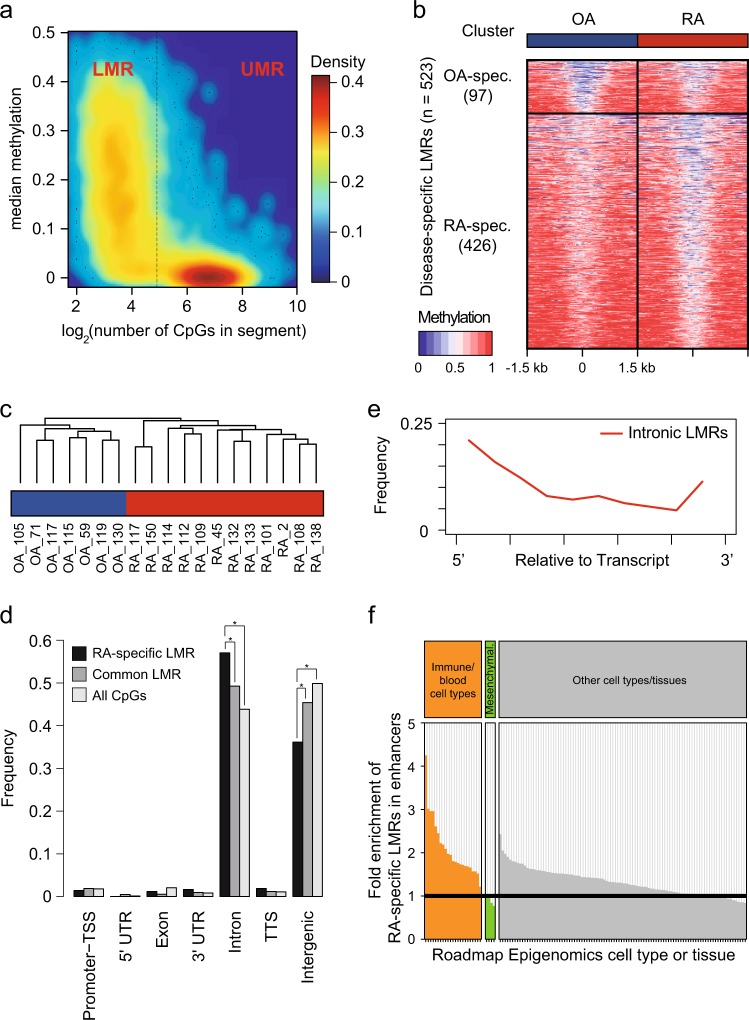
Identification of focal methylated regions specific to RA or OA. **a** A colored scatterplot showing hypomethylated regions after excluding PMDs. The middle line divides the regions into unmethylated regions (UMRs, left) and low-methylated regions (LMRs, right). Densities of individual CpGs are estimated using kernel functions and are colored-scaled. **b** A heatmap with clusters of LMRs specific to each disease. Ticks represent distance from the center of given LMRs. **c** A hierarchical clustering of samples based on the Jaccard indexes calculated from LMRs. OA is blue and RA is red. **d** The distribution of intragenic LMRs. *P < 0.01, Pearson’s chi-squared test. **e** The distribution of intronic RA-specific LMRs relative to transcripts. **f** A bar plot with the ratio of RA-specific LMRs to OA-specific LMRs overlapping enhancers in blood/immune cell types (left), mesenchymal cell types (middle), and other cell types (right). Enhancers were inferred from the chromatin landscape of NIH’s Roadmap Epigenomics project^29^

Notably, more than 3.5 million CpGs (17.0%) exhibited a low to intermediate level of methylation in the range from 0.1 to 0.5 (Supplementary Figure S1b). Clusters of this population generated distinct focal regions termed localized LMRs. After excluding PMDs, a hidden Markov model was applied to identify UMRs and LMRs in individual samples (Fig. 2a). To define RA- or OA-specific LMRs, all detected LMRs were divided by the k-means clustering method. Finally, 523 disease-specific LMRs were identified (426 for RA, 97 for OA) (Fig. 2b, Supplementary Table S3). Although methylation in LMRs was generally lower than surrounding regions, the difference between RA and OA was obvious. A hierarchical clustering of the Jaccard index clearly grouped RA samples different from OA samples, indicating that LMRs are a signature of RA and OA (Fig. 2c).

Approximately half of the RA-specific LMRs were located in genic regions (Fig. 2d). Many genic LMRs were located in intron regions. They were preferentially positioned at the 5′ ends along genes (Fig. 2e). For instance, 32.8% (78/238) of intronic LMRs were detected in the first introns. Compared to OA-specific LMRs, RA-specific LMRs were enriched at enhancers of immune or blood cell types but depleted at enhancers of mesenchymal cell types (Fig. 2f, Supplementary Table S3). These results suggested that RA-specific LMRs most likely act as distal regulatory elements for immune responses.

**Fig. 3:**
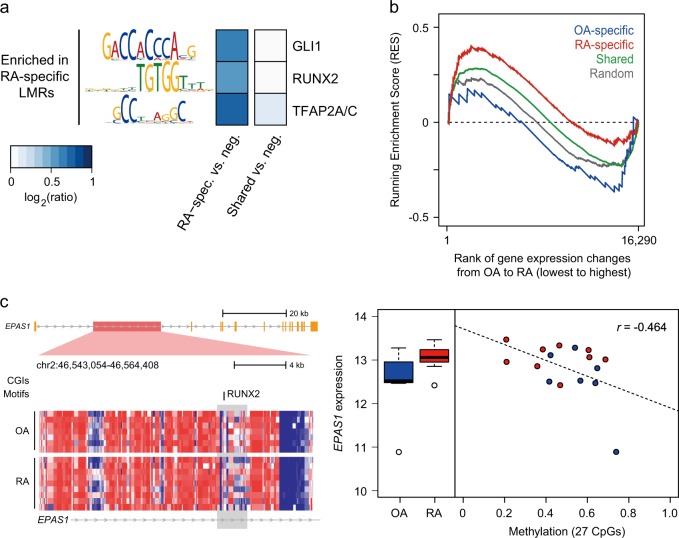
Methylation in LMRs and expression of downstream targets. **a** A heatmap with enriched transcription factor binding motifs in LMRs. **b** A line plot showing the relationship between LMRs and downstream targets. LMRs specific to a certain disease were highly enriched with the highly expressed genes in a certain disease. Downstream targets were chosen from genes with LMRs or were located within the downstream 5 kb from LMRs. Random represents permutated data. **c** An example of *EPAS1* with an RA-specific LMR. A left heatmap describes methylation in regions around an LMR. Methylation is colored from blue (low) to red (high). The LMRs are marked with a gray box. In the LMR, methylation is generally lower in RA than OA. A right plot depicts methylation in an LMR in each disease (left) and individual samples (right). Hypomethylation of the LMR is coupled with increased expression of this gene in RA (red) compared to OA (blue). RES Running Enrichment Score

DNA-binding proteins, especially TFs, have their own binding motifs or consensus sequences. Three known motifs (GLI1, RUNX2, and TFAP2A/C) were sequence features enriched in RA-specific LMRs. Control regions were selected from sequences that had the same genomic distribution as LMRs but were not LMRs (Fig. 3a). These motifs found in RA-specific LMSs were not detected in OA-specific LMRs. Moreover, no motif was discovered as common in LMRs between RA and OA. Next, we explored the potential functional role of LMRs as *cis*-regulatory elements. By matching LMRs to the nearest genes, the GSEA revealed that RA-specific LMRs were highly enriched in the RA upregulated genes (Fig. 3b, Running Enrichment Score (RES) = 1.47, FDR *q*-value = 0.009). In contrast, OA-specific LMRs showed negative enrichment (RES = −1.10, FDR *q*-value = 0.166). Among RA-specific LMRs allocated to the nearest genes, the methylation of 21 LMRs showed significant correlations with expression of the matched genes (*P* < 0.05, 20 negative, 1 positive, Supplementary Table S4). For example, an LMR detected only in RA samples was located in the first intron of the endothelial PAS domain-containing protein 1 (*EPAS1*) gene, often known as hypoxia inducible factor 2 (HIF2A), which is a pivotal component in RA pathogenesis^34^ (Fig. 3c). Hypomethylation of this LMR in RA is coupled with increased expression of *EPAS1*. These results suggest that LMRs may be crucial factors showing the difference between RA and OA.

### Differentially variable and DMCs

**Fig. 4:**
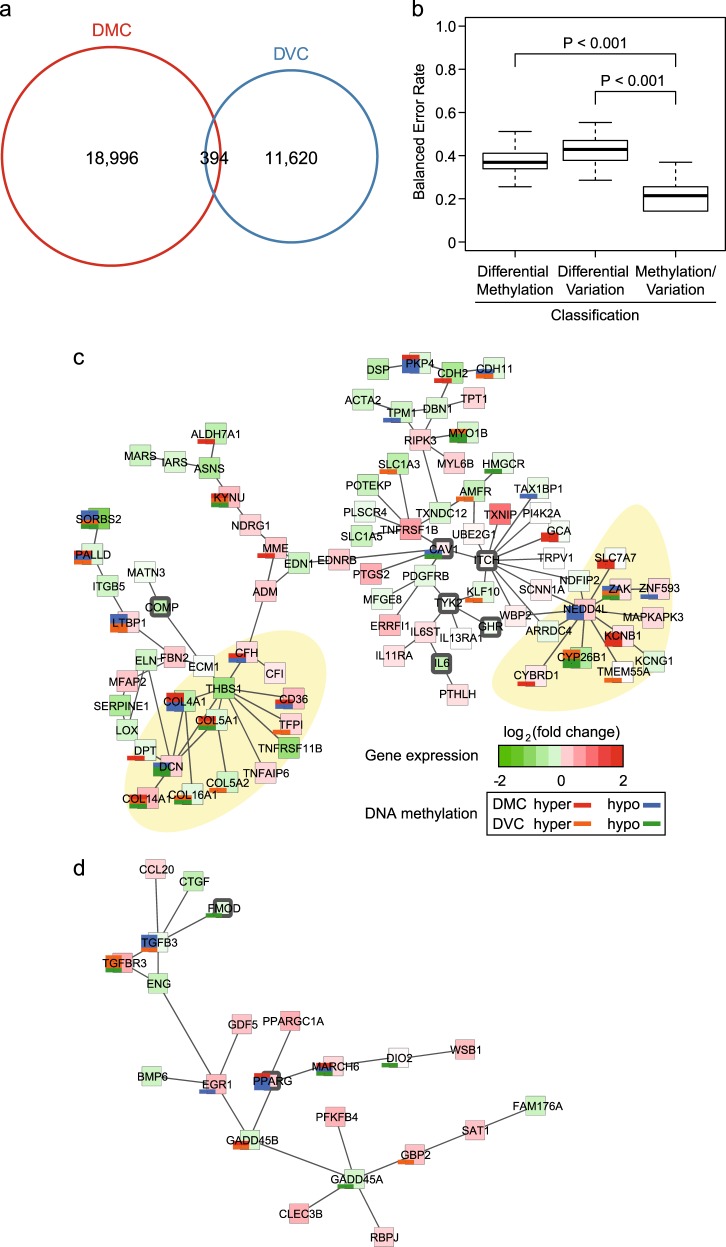
Single base-scale methylated positions with average and variability. **a** Venn diagrams with the number of differentially methylated CpGs (DMCs) and differentially variable CpGs (DVCs). **b** The distribution of balanced error rates of classification using DMCs or DVCs. Significance levels were estimated from permutation test with 999 Monte Carlo replications. **c** The largest subnetwork is linked to response to wounding, tissue development, and collagen fibril organization. Yellow areas denote where DMCs or DVCs are concentrated. Colors in large boxes represent gene expression levels in RA relative to OA. Red is upregulated, and green is downregulated. Stacked bar plots represent the number of DMCs or DVCs in certain genes. DMCs are colored red (hypermethylated) and blue (hypomethylated), while DVCs are colored orange (hypervariable) and green (hypovariable). **d** The second largest subnetwork is related to TGF-β receptor signaling

Single base-scale methylated CpGs can be valuable as easy-to-measure biomarkers for assessing the status of a disease or medical condition. There were 19,390 DMCs (11,058 hypermethylated and 8332 hypomethylated) in RA compared to OA (Supplementary Table S5). Additionally, DVCs (5570 hypervariable and 6444 hypovariable) in RA compared to OA were identified (Supplementary Table S5). Out of DMCs and DVCs, 394 CpGs were significantly overlapped (Fig. 4a, Fisher’s exact test with *P* < 2.2 × 10^−16^, permutation test with *P* < 0.001). Considering both DMC and DVC together demonstrated a lower balanced error rate of classification than when considering only one of them, suggesting that they are in complementary relations for classification (Fig. 4b).

To test whether there was pathogenic disease relevance with DMC or DVC, a combinatorial analysis of single base-scale methylated CpGs and gene expression profiles was performed. This analysis led to the identification of six significant subnetworks using PhenomeExpress^35^ (Table 1, Fig. 4c, d, Supplementary Figure S4). For example, the first subnetwork was related with wound response, tissue development and collagen fibril organization (Fig. 4c). DMCs and DVCs were highly concentrated in genes encoding collagen proteins and proteins interacting with NEDD4L, an E3 ubiquitin protein ligase. The second subnetwork harbored genes associated with the transforming growth factor beta (TGF-β) receptor signaling pathway, in which DMCs and DVCs were also detected in hub genes (Fig. 4d).

**Table 1:** Significantly enriched biological functions in each subnetwork

Network no.	No. of nodes	Empirical *P* value	Description
1	89	1.52E-08	Response to wounding
		4.16E-08	Tissue development
		2.55E-07	Collagen fibril organization
2	6	3.11E-05	Regulation of cell differentiation
3	22	1.24E-08	Transforming growth factor beta receptor signaling pathway
4	6	1.75E-04	Microtubule-based movement
5	7	4.74E-05	Negative regulation of transport
6	5	6.98E-04	Negative regulation of signal transduction

### MicroRNAs and DNA methylation

**Fig. 5:**
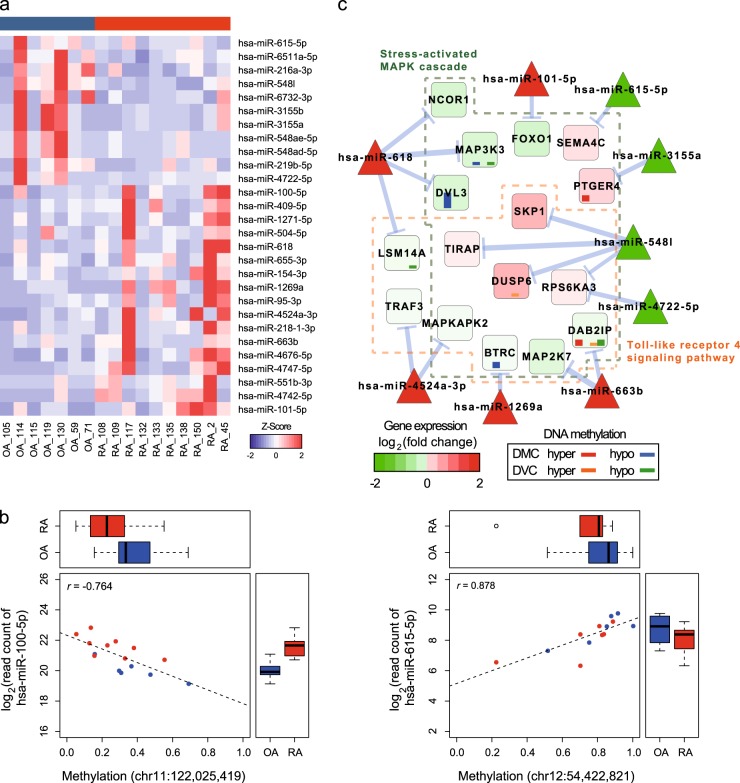
Identification of differentially expressed miRNAs between RA and OA. **a** A heatmap with miRNAs identified as differentially expressed. Raw counts of the miRNAs were transformed into *Z*-scores and color scaled. **b** Scatterplots describe methylation in the single CpGs and miRNAs expression in individual samples. Box plots show methylation (top) and miRNA expression in each disease (right). **c** A subnetwork of microRNA–mRNA pairs. Colors in circles represent gene expression levels in RA relative to OA. Red is upregulated and green is downregulated. Blue arrows average negative correlations. Inner bar plots represent the number of DMCs or DVCs in certain genes. DMCs are colored red (hypermethylated) and blue (hypomethylated), while DVCs are colored orange (hypervariable) and green (hypovariable)

After filtering miRNA sequencing data, ten RA and seven OA samples were selected and further examined. Sequencing results are summarized in Supplementary Table S2. mirDeep2 (ref. ^36^) was used to identify differentially expressed miRNAs (DEmiRs) between RA and OA. Thirty-nine DEmiRs were found with a fold change >2 and unadjusted *P* < 0.05. Among them, 28 miRNAs were expressed in at least 50% of samples (Fig. 5a, Supplementary Table S6). Then, miRNA expression was compared to single base-scale CpG methylation. With absolute Spearman correlation coefficient >0.5 and permutation *P* < 0.05, the comparison showed that 201 CpGs were significantly correlated with 27 miRNAs located near the CpGs (Fig. 5b, Supplementary Table S6).

Using previously predicted miRNA–mRNA pairs, novel target genes of these 27 miRNAs were investigated. As expected, negative Spearman correlation coefficients were shown in 227 pairs with 16 miRNAs (permutation *P* < 0.05) (Supplementary Figure S5, Supplementary Table S6). From the list of miRNAs and genes, gene ontology and pathway annotation network analysis by ClueGO^37^ provided a gene subnetwork of stress-activated MAPK cascade and toll-like receptor 4 (TLR4) signaling pathway, where 43.8% (7/16) of target genes contained DMCs or DVCs (Fig. 5c).

### Rare genetic variants irrelevant to DNA methylation

Whole-exome sequencing analysis was used to identify the genetic variations by examining all protein-coding sequences. Eleven RA and seven OA samples were selected to identify rare genetic variants specific to RA or OA. The sequencing results exhibited 92.2× coverage and covered 94.7% of exomes (≥20×) (Supplementary Table S2). There was no obvious difference in mutation rates between RA and OA (deleterious variation, *P* = 0.67; loss of function mutation (nonsense, splice site, frameshift), *P* = 0.15; missense mutation, *P* = 0.80). In total, 97 genes with sequence variations detected in at least two RA samples but not in OA samples were identified.

**Fig. 6:**
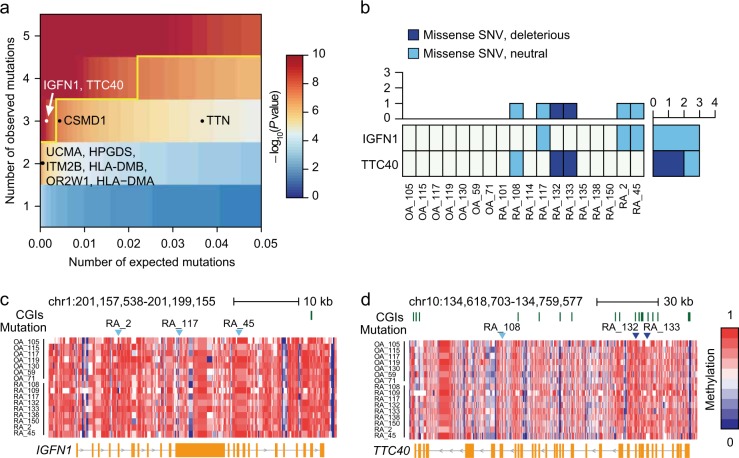
Identification of significant rare genetic variants. By analyzing RA and OA exome sequencing data, sites and frequency of variants were identified. **a** A heatmap showing significance levels of detected variants. Degrees of significance levels are color scaled. White points are significant variants, and black points are not significant. A yellow line describes the cutoff. **b** A heatmap showing the number of variants in each sample. **c** The methylation profile along the *IGFN1* gene locus is shown color scaled from blue (low) to red (high). CpG islands (CGIs) are marked by green ticks above the heatmap. Sites of variants are shown in blue triangles. **d** The chromosomal position, CGI location, sites of variants, and methylation profile are depicted in the *TTC40* gene locus

Recently, Samocha et al.^31^ developed a framework for the interpretation of de novo mutations by distinguishing disease-related mutations from background variations. Applying Samocha’s model to our exome data revealed that, unlike OA, there were significant mutations in RA (conservative significance threshold (*P* = 1 × 10^−8^)) (Fig. 6a). *IGFN1* and *TTC40* had more mutations than predicted. All mutations in *IGFN1* were missense but neutral (Fig. 6b). A variant in *TTC40* was missense and neutral, whereas two variants were missense and deleterious. However, the *TTN* gene with the longest coding sequence was not significant because the number of observed mutations was below the cutoff value. Similarly, genes such as *HLA-DMA, HLA-DMB UCMA*, *HPGDS*, *ITM2B*, and *OR2W1* did not exhibit significant de novo mutations. The relationship between de novo mutations in RA and DNA methylation was not correlated (Fig. 6c, d).

## Discussion

DNA methylation plays an important role in the pathogenesis of RA^15–17^. However, genome-wide studies for DNA methylation in RA have mainly been based on the DNA microarray technique, and the results cover only a subset of the genome as limited by probes. Here, we reported the first investigation of whole-genome DNA methylation of synoviocytes from RA and OA patients through WGBS technology. The WGBS results demonstrate comprehensive genomic coverage, high quantitative accuracy, and outstanding reproducibility^19^. In our data, the overall distribution of DNA methylation was almost similar between RA and OA, which is in discord with previous observations showing prominent global hypomethylation in RA synoviocytes^38,39^. The methylation patterns were too heterogeneous among individual samples even in the same disease to identify specific large-scale regions. However, we could find distinct focal regions termed LMRs with hundreds of base pairs. The methylation profiles of LMRs could clearly distinguish RA from OA. Approximately half of LMRs were located in genic regions, particularly in the introns at 5′ ends. This finding is consistent with the previous finding that the first introns within most genes are important in transcription and subject to easy epigenetic modification, association with TFs, and open chromatin structure formation^40^. Compared to OA-specific LMRs, RA-specific LMRs were enriched in enhancers of immune or blood cell types but depleted in enhancers of mesenchymal cell types. Fibroblast-like synoviocytes in joints originate from mesenchymal cells. However, the synoviocytes from RA patients show altered phenotypes compared to the cells in normal tissue^41^. The presence of different LMRs between RA and OA reflects functional changes of synoviocytes in patients and supports their importance as regulatory elements for the particular immunological functions in RA.

Some RA-specific LMRs overlapped with specific motifs of TFs such as GLI1, RUNX2, and TFAP2A/C. These TFs are closely related to the TGF-β pathway^42–44^. GLI1 is a zinc-finger protein and mediates Sonic hedgehog (Shh) signaling. A recent study observed that activated Shh signaling drives proliferation of synoviocytes in RA^45^. It is known that Shh signaling is involved in cartilage damage in RA^46^. RUNX2 is essential for skeletal development, and its known target genes are osteopontin, collagenenase 3, and VEGF, which are engaged in the RA pathogenesis^47^. A previous study observed that RUNX2 is important in the development and migration of plasmacytoid dendritic cells^48^, which can be recruited to RA synovial tissue^49^. However, little is known about the role of TFAP2A/C in RA, and a further study will help us to understand its role. Therefore, it is plausible that DNA methylation is related with RA in connection with TFs.

One of the fundamental roles of DNA methylation is transcriptional regulation. In our data, some LMRs specific to RA were highly linked with relatively upregulated genes. For example, HIF-2α, the product of the *EPAS1* gene, plays a pivotal role in the pathogenesis of RA^34^. *GPC6* has been identified in a genome-wide association analysis^50^ and shown to be differentially expressed in synovium with chronic inflammation^51^. *LTBP1* is important in the TGF-β pathway in RA synoviocytes^52^, and *NDRG1* is necessary for apoptosis signaling^53^. Because LMRs act as distal regulatory regions or TFs^20,25^, additional distant genes influenced by the LMRs can be identified by a technique to capture long-range chromatin interactions^54,55^.

A few studies have raised the possibility of single base-scale methylated CpGs as biomarkers in multiple cases^14,56,57^. As a biomarker, single CpG methylation can be more easily detected than other types of information such as proteins, metabolites, etc. In addition, several studies have mentioned the potential of CpGs with differential variable methylation as good biomarkers^33,58–60^. Our RA-specific DMCs and DVCs may also be potential biomarkers. A combination of single base-scale methylated CpGs and gene expression profiles could help to understand pathogenic mechanisms of RA. In disease-relevant subnetworks, DMCs or DVCs were located in genes encoding collagen, NEDD4L-related molecules, and TGF-β-related molecules, specifically. These results suggest that those single CpGs could participate in the regulation of RA-relevant genes.

Among 28 differentially expressed miRNAs (DEmiRs) between RA and OA, 27 miRNAs showed significant correlations with DNA methylation at the single CpG level. It suggests that single CpGs may also have a potential role in the regulation of miRNAs. In this study, we identified novel target genes of dysregulated miRNAs in RA. Functionally organized subnetworks of miRNAs and mRNAs were closely related to biological functions such as the stress-activated MAPK cascade and TLR4 signaling pathway. *TLR2* and *TLR4* are highly expressed in RA synovial fibroblasts and probably contribute to the destructive phase of RA^61^. A group of DEmiR target genes with DMCs or DVCs was identified in the subnetworks. These genes might be potentially regulated by DNA methylation at the transcriptional level and targeted by miRNAs at the post-transcriptional level.

An approach to analyze DNA methylation together with rare genetic variants is quite different from other epigenome-wide association studies^14,16,62^ that attempt to link epigenetic changes with common genetic variants. Consequently, our approach enabled detection of genes associated with rare genetic variants by applying Samocha’s statistical framework^31^. Following this statistical analysis for identification of de novo mutations, we obtained confident results by excluding false-positive genes. For instance, *TTN* is a known false positive in many studies due to its long coding sequence^31,63^. Furthermore, polymorphisms of the *DMA* and *DMB* genes do not influence susceptibility to develop RA^64^. Rare genetic variants identified in RA may be independent of DNA methylation. Moreover, *IGFN1* and *TTC40* were unidentified in gene expression data (Supplementary Table S4). Therefore, their role in the pathogenesis of RA is not clear at this moment; thus, their biological significance should be further examined by an in-depth study.

In conclusion, we identified the epigenetic features in RA and their roles in transcriptional regulation. In particular, our research focused on DNA methylation and miRNA profiles. Despite the intrinsic heterogeneity in RA samples^6,7^, the comparative DNA methylation analysis at multiple layers with PMD, LMR, and DMC/DVC provided a way to interpret DNA methylation profiles to understand the disease pathogenesis. Additionally, our data of DNA methylation, miRNA, gene expression, and rare genetic variants generated from the same synoviocytes of RA or OA patients could provide valuable resources for developing biomarkers and key regulators related to the pathological phenotype of RA.

## Supplementary information

Supplementary Information

Figure S1

Figure S2

Figure S3

Figure S4

Figure S5

Supplementary Table S1

Supplementary Table S2

Supplementary Table S3

Supplementary Table S4

Supplementary Table S5

Supplementary Table S6

## Data Availability

All data are accessible at the GATEONE system of Korea National Institutes of Health via virtual private network (VPN, https://152.99.73.2) upon request. Regarding the usage and analysis of data downloaded from the GATEONE, we obtained IRB review exemption on human-derived materials from POSTECH (PIRB-201E085).
