# Supplementary material for: Epigenetic analysis in rheumatoid arthritis synoviocytes
Source: Exp Mol Med. 2019 Feb 22;51(2):22. doi: 10.1038/s12276-019-0215-5 (PMC6395697; doi:10.1038/s12276-019-0215-5)
Supplement: Supplementary file 1 — Supplementary Information [file 12276_2019_215_MOESM1_ESM.docx]

Supplementary Information

**Epigenetic analysis in rheumatoid arthritis synoviocytes**

Seokjin Ham^1^, Jae-Bum Bae^2^, Suman Lee^2^, Bong-Jo Kim^2^, Bok-Ghee Han^2^, Seung-Ki Kwok^3^, Tae-Young Roh^1, 4^

^1^Department of Life Sciences, ^4^Division of Integrative Biosciences and Biotechnology, POSTECH, Pohang 37674, Korea, ^2^Division of Genome Research, Center for Genome Science, Korea National Institute of Health, Korea Centers for Disease Control and Prevention, Osong 28160, Korea, ^3^Division of Rheumatology, Department of Internal Medicine, Seoul St. Mary's Hospital, College of Medicine, The Catholic University of Korea, Seoul 06591, Korea

**Short Running Head**: Epigenetic analysis in RA synoviocytes

**Supplementary materials and methods**

**Gene expression microarray**

Samples were prepared and analyzed with Human HT-12 v4 Expression BeadChip Kit (Illumina, USA) following procedures as a manufacturer provided. Image data was processed by BeadStudio 2.0 (Illumina, USA). Gene expression data was additionally analyzed using lumi^1^ Bioconductor package. Probes were processed through variance stabilizing transform, robust spine normalization (RSN), and quality control. A filtering step removed sites with detection P < 0.05 in more than four samples. Statistical tests were performed by limma^2^ Bioconductor package.

**450K Infinium methylation array**

Samples were prepared and processed with Infinium HumanMethylation450 BeadChip Kit (Illumina, USA) following procedures as a manufacturer provided. Image data was processed by BeadStudio 2.0 (Illumina, USA). Infinium data was analyzed using lumi^1^ and methylumi^3^ Bioconductor packages. Loci on autosomes were retained, but sites with any sample with P > 0.01 were excluded. Data was processed by color balance adjustment, background level correction, and quantile normalization. Sites overlapping with known single nucleotide polymorphisms (SNPs) in dbSNP 142 or those predicted to cross-hybridize with the sex chromosomes were filtered out. If 47 of 50 nucleotides in a given probe were matched to the sex chromosome sequence, that locus was considered as cross-hybridization and removed. Methylation in the remaining probes were additionally normalized using BMIQ^4^ procedure.

**Whole-genome bisulphite sequencing**

WGBS data was analyzed using Bismark version 0.14.0^5^. Alignment was performed with the non-default parameter -n 1 onto the hg19 genome (GRCh37). Duplicate reads were then removed. Methylation extraction was performed with the non-default parameters -p --no_overlap --bedGraph --counts --cytosine_report --gzip --split_by_chromosome. A methylation bias plot assessed the magnitude of introduced constitutively unmethylated Cs and it showed constant methylation levels about 0.7 along the reads in all samples.

Subsequently, information from both strands was combined and the percentage of genomic CpGs covered by more than five, ten, and twenty reads was calculated (Supplementary Table S2). Methylation of appropriate internal control markers in *N4BP2* gene was examined as previously described^6^. Then, CpGs on sex chromosomes and CpGs with a coverage of less than four reads over 80% of samples were filtered out. Concordance of methylation between WGBS data and Infinium data was very high (γ = 0.942 ± 0.01).

Average methylation levels and standard deviations of individual CpGs were compared between RA and OA. Scatter plots with different colors were generated using fields R package. Two-sample Kolmogorov-Smirnov test was also performed in R. Average methylation levels and CpG densities within fixed windows of 100 bp were calculated using Repitools^7^ Bioconductor package. Box plots displaying the distribution of average methylation rate by each CpG density were generated in R using default settings. A heatmap with hierarchical clustering was generated with the top 500 CpGs with the highest variability in R. Another heatmap was plotted using recursively partitioned mixture model (RPMM) as previously described^8^. The information of annotations in hg19 genome was obtained from the Table Brower in the UCSC genome browser (genome.ucsc.edu).

**Partially methylated domains (PMDs)**

PMDs for each sample were defined in a similar manner as previously described.^9^ All steps proceeded with MethylSeekR^9^ Bioconductor package. Average methylation within sliding windows of 100 consecutive CpGs was calculated and the distribution of α-values was estimated from the typical polarized distribution of methylation. Then, hidden Markov model (HMM) identified PMDs. Methylation plots were generated with fixed windows of 100 consecutive CpGs using the GenomicRanges^10^ Bioconductor package.

**Locally low methylated regions (LMRs)**

After masking PMDs from genome, segmentation of the remaining regions was performed using MethylSeekR Bioconductor package as previous described^9^. Methylation levels were smoothed over three consecutive CpGs and hypomethylated regions are identified with consideration for the fixed cutoff 0.5 and the minimal number of four CpGs. They were determined at false discovery rate (FDR) below 5%, which was estimated based on the randomization of methylation levels outside of CpG islands. Identified hypomethylated regions were then classified into unmethylated regions (UMRs) and LMRs with a cutoff of thirty CpGs they have (UMRs ≥ 30, LMRs < 30).

Methylation levels around 1.5 kb from the center of LMRs were calculated and used for dissection of LMRs by k-means clustering in R. Jaccard index was computed for measuring similarities between LMRs in any two samples. It was the ratio of the combined length of the genomic regions within LMRs in both samples and in at least one sample. Hierarchical clustering with the Jaccard index was carried out in R. Genomic annotations of LMRs was performed using HOMER version 4.7.2^11^. Significance was calculated using Pearson's chi-squared test in R. Locations of all enhancers inferred from chromatin landscapes^12^ was compared to locations of LMRs.

To identify enriched sequence features, sequences around 100 bp from the center of specific LMRs were divided into five subsets. For *de novo* motif discovery, two subsets were used by DREME tool (part of the MEME suite, version 4.10.0^13^) with the default settings. As a negative control, the regions that had the same genomic distribution, but not with LMRs were used. Motifs suggested were integrated with the known motifs in database given by MEME suite. To select proper motifs, position weight matrices of these motifs were mapped against another two subsets using the FIMO tool (part of the MEME suite) with the parameter --thresh 9.76 x 10^-4^ (4 x 1/4^6^). The number of occurrences of a given motif in specific LMRs was compared to that in negative control or shared LMRs. Only motifs showing P < 0.05 were selected as significant motifs and P was calculated using Pearson's chi-squared test. Whether these motifs were repetitively shown in other specific LMRs, the same analysis above was performed with the remaining subset. Motifs showing P < 0.05 in this step were finally selected as motifs significantly enriched in specific LMRs.

Downstream targets were chosen from genes which contained LMRs or were located within downstream 5 kb from LMRs. The relationship between specific LMRs and downstream targets was tested adapting the gene set enrichment analysis (GSEA)^14^. Genes were ordered by the signal to noise ratio (S/N) in expression between RA and OA. The order is related with gene expression levels in RA compared to OA. The running enrichment score was calculated by occurrence of LMRs associated with downstream targets, allowing multiple LMRs per gene. The calculation was weighted by the S/N. The maximum running motif enrichment value was finally used. P and FDR was calculated from a permutation test. Randomized data was generated from permutated data. Motif enrichments in LMRs associated with downstream targets were analyzed in the same way. With absolute Pearson correlation value > 0.5 and permutation P < 0.05, direct comparisons between LMRs and target genes were identified. Methylation plots were generated using the Gviz^15^ Bioconductor package.

**Differentially methylated CpGs (DMCs) and differentially variable CpGs (DVCs)**

To identify DMCs between RA and OA, logistic regression in MethylKit^16^ was applied with methylation difference > 0.2 and P < 2.42 x 10^-9^ (0.05/20,605,641, Bonferroni correction). For verification, CpGs commonly detected in both WGBS data and Infinium data were selected among DMCs.

To identify DVCs between RA and OA, Levene's test by missMethyl^17^ was performed with FDR < 0.05. Significance of the overlap between DMCs and DVCs were determined by Fisher’s exact test with P < 2.2 x 10^-16^ and permutation test with P < 0.001 in R. Measuring a balanced error rate of the prediction with DMCs or DVCs was carried out using ClassifyR^18^ Bioconductor package with default parameters. Significance levels were estimated from 999 Monte Carlo replications using perm package.

**Disease-related subnetworks**

The PhenomeExpress^19^ was used to build a vital subnetworks in RA and OA and identify core disease pathways. It required transcriptome data, protein-protein interactions (PPI) and phenotype similarity networks. Transcriptome data was used from gene expression microarray previously mentioned. PPI network was given by the program which included Human ConsensusPathDB^20^ and STRING database^21^. Phenotype-gene associations were inferred from UberPheno cross-species ontology.^22^ Semantic similarity between the phenotype terms was calculated using the FastSemSim version 0.9.4 Python package with a cutoff of 0.9. The output format could be accessed by Cytoscape version 3.2.1 (www.cytoscape.org). BiNGO version 2.44^23^ was used to assess significantly enriched functions in each subnetwork.

**miRNA sequencing**

Small RNA sequencing data were analyzed using miRDeep2 version 0.0.7^24^. Adaptor-trimmed reads were aligned to the hg19 reference genome and then further to predefined miRNA precursors sequences of miRBase version 21^25^. Expression of mature miRNAs was determined relative to all aligned reads. Differentially expressed miRNAs were identified using the DESeq^26^ Bioconductor package with fold change > 2 and unadjusted P < 0.05. Then, miRNAs expressed (the raw read counts > 0) in at least 50% of samples were selected. DNA methylation levels of the loci within upstream 5 kb or gene bodies of miRNAs were compared to expression levels of the miRNAs. With absolute Spearman correlation value > 0.5 and permutation P < 0.05, significant relations were identified.

To identify functionally important miRNAs, the correlation between miRNAs and target genes was examined. Expression of miRNAs was normalized based on size factors estimated by the DESeq^26^. Previously predicted target relations were obtained by merging the TargetScan 7.0^27^, PicTar 2^28^, PITA 6^29^, microRNA.org^30^, miRDB 5.0^31^, miRecords^32^, RNA22-HSA v2.0^33^, TargetMiner^34^ databases. Among them, the confident relations found in more than four databases were selected. With negative Spearman correlation value with permutation P < 0.05, significant relations were identified. The output format could be accessed by Cytoscape version 3.2.1 (www.cytoscape.org). From the lists of genes, ClueGO 2.2.3^35^ integrated Gene Ontology (GO) terms and created functionally organized subnetworks with P < 0.05.

**Exome sequencing**

Rare genetic variants for each sample were detected using TREVA in a similar fashion to that previously described^36^. In brief, TREVA aligned reads onto hg19 human genome, detected PCR duplicates, realigned reads around INDEL and recalibrate base qualities. After calling genetic variants, it annotated them using ENSEMBL database and predicted their functions. Following thresholds were used in filtering steps: in autosomes, in canonical transcripts, with the most deleterious prediction consequence, with Phred quality ≥ 30, read depth ≥ 10, not known in dbSNP 142, with minor allele frequency ≤ 0.01 in 1000 Genome Asian Phase I, with alternative allele < 5% of the reads in the other disease (RA or OA), normalized score ≥ 2, homopolymer run ≤ 5, strand bias ≤ 60, and finally with deleterious consequences.

To assess whether the observed variants were disease-relevant or background variation, a recently devised statistical framework^37^ was applied. A conservative significance threshold (P = 1.0 x 10^-8^) was applied to get significant variants. Methylation plots with candidate genes were generated using the Gviz^15^ Bioconductor package.

**References**

1 Du P, Kibbe WA and Lin SM. lumi: a pipeline for processing Illumina microarray. *Bioinformatics* 2008; **24**: 1547-8

2 Ritchie ME, Phipson B, Wu D, Hu Y, Law CW, Shi W, et al. limma powers differential expression analyses for RNA-sequencing and microarray studies. *Nucleic Acids Res* 2015; **43**: e47

3 Davis S, Du P, Bilke S, Triche TJ and Bootwalla M. methylumi: Handle Illumina methylation data. R package, 2015

4 Teschendorff AE, Marabita F, Lechner M, Bartlett T, Tegner J, Gomez-Cabrero D, et al. A beta-mixture quantile normalization method for correcting probe design bias in Illumina Infinium 450 k DNA methylation data. *Bioinformatics* 2013; **29**: 189-96

5 Krueger F and Andrews SR. Bismark: a flexible aligner and methylation caller for Bisulfite-Seq applications. *Bioinformatics* 2011; **27**: 1571-2

6 Lu TP, Chen KT, Tsai MH, Kuo KT, Hsiao CK, Lai LC, et al. Identification of genes with consistent methylation levels across different human tissues. *Sci Rep* 2014; **4**: 4351

7 Statham AL, Strbenac D, Coolen MW, Stirzaker C, Clark SJ and Robinson MD. Repitools: an R package for the analysis of enrichment-based epigenomic data. *Bioinformatics* 2010; **26**: 1662-3

8 Houseman EA, Christensen BC, Yeh RF, Marsit CJ, Karagas MR, Wrensch M, et al. Model-based clustering of DNA methylation array data: a recursive-partitioning algorithm for high-dimensional data arising as a mixture of beta distributions. *BMC Bioinformatics* 2008; **9**: 365

9 Burger L, Gaidatzis D, Schubeler D and Stadler MB. Identification of active regulatory regions from DNA methylation data. *Nucleic Acids Res* 2013; **41**: e155

10 Lawrence M, Huber W, Pages H, Aboyoun P, Carlson M, Gentleman R, et al. Software for computing and annotating genomic ranges. *PLoS Comput Biol* 2013; **9**: e1003118

11 Heinz S, Benner C, Spann N, Bertolino E, Lin YC, Laslo P, et al. Simple combinations of lineage-determining transcription factors prime cis-regulatory elements required for macrophage and B cell identities. *Mol Cell* 2010; **38**: 576-89

12 Roadmap Epigenomics C, Kundaje A, Meuleman W, Ernst J, Bilenky M, Yen A, et al. Integrative analysis of 111 reference human epigenomes. *Nature* 2015; **518**: 317-30

13 Bailey TL, Boden M, Buske FA, Frith M, Grant CE, Clementi L, et al. MEME SUITE: tools for motif discovery and searching. *Nucleic Acids Res* 2009; **37**: W202-8

14 Jolma A, Yan J, Whitington T, Toivonen J, Nitta KR, Rastas P, et al. DNA-binding specificities of human transcription factors. *Cell* 2013; **152**: 327-39

15 Hahne F, Durinck S, Ivanek R, Mueller A, Lianoglou S, Tan G, et al. Gviz: Plotting data and annotation information along genomic coordinates. In R packageed^eds), 2015

16 Akalin A, Kormaksson M, Li S, Garrett-Bakelman FE, Figueroa ME, Melnick A, et al. methylKit: a comprehensive R package for the analysis of genome-wide DNA methylation profiles. *Genome Biol* 2012; **13**: R87

17 Phipson B and Oshlack A. DiffVar: a new method for detecting differential variability with application to methylation in cancer and aging. *Genome Biol* 2014; **15**: 465

18 Strbenac D, Mann GJ, Ormerod JT and Yang JY. ClassifyR: an R package for performance assessment of classification with applications to transcriptomics. *Bioinformatics* 2015; **31**: 1851-3

19 Soul J, Hardingham TE, Boot-Handford RP and Schwartz JM. PhenomeExpress: a refined network analysis of expression datasets by inclusion of known disease phenotypes. *Sci Rep* 2015; **5**: 8117

20 Kamburov A, Stelzl U, Lehrach H and Herwig R. The ConsensusPathDB interaction database: 2013 update. *Nucleic Acids Res* 2013; **41**: D793-800

21 Szklarczyk D, Franceschini A, Kuhn M, Simonovic M, Roth A, Minguez P, et al. The STRING database in 2011: functional interaction networks of proteins, globally integrated and scored. *Nucleic Acids Res* 2011; **39**: D561-8

22 Kohler S, Doelken SC, Ruef BJ, Bauer S, Washington N, Westerfield M, et al. Construction and accessibility of a cross-species phenotype ontology along with gene annotations for biomedical research. *F1000Res* 2013; **2**: 30

23 Maere S, Heymans K and Kuiper M. BiNGO: a Cytoscape plugin to assess overrepresentation of gene ontology categories in biological networks. *Bioinformatics* 2005; **21**: 3448-9

24 Friedlander MR, Mackowiak SD, Li N, Chen W and Rajewsky N. miRDeep2 accurately identifies known and hundreds of novel microRNA genes in seven animal clades. *Nucleic Acids Res* 2012; **40**: 37-52

25 Kozomara A and Griffiths-Jones S. miRBase: integrating microRNA annotation and deep-sequencing data. *Nucleic Acids Res* 2011; **39**: D152-7

26 Anders S and Huber W. Differential expression analysis for sequence count data. *Genome Biol* 2010; **11**: R106

27 Agarwal V, Bell GW, Nam JW and Bartel DP. Predicting effective microRNA target sites in mammalian mRNAs. *Elife* 2015; **4**. doi: 10.7554/eLife.05005.

28 Blin K, Dieterich C, Wurmus R, Rajewsky N, Landthaler M and Akalin A. DoRiNA 2.0--upgrading the doRiNA database of RNA interactions in post-transcriptional regulation. *Nucleic Acids Res* 2015; **43**: D160-7

29 Kertesz M, Iovino N, Unnerstall U, Gaul U and Segal E. The role of site accessibility in microRNA target recognition. *Nat Genet* 2007; **39**: 1278-84

30 Betel D, Wilson M, Gabow A, Marks DS and Sander C. The microRNA.org resource: targets and expression. *Nucleic Acids Res* 2008; **36**: D149-53

31 Wong N and Wang X. miRDB: an online resource for microRNA target prediction and functional annotations. *Nucleic Acids Res* 2015; **43**: D146-52

32 Xiao F, Zuo Z, Cai G, Kang S, Gao X and Li T. miRecords: an integrated resource for microRNA-target interactions. *Nucleic Acids Res* 2009; **37**: D105-10

33 Miranda KC, Huynh T, Tay Y, Ang YS, Tam WL, Thomson AM, et al. A pattern-based method for the identification of MicroRNA binding sites and their corresponding heteroduplexes. *Cell* 2006; **126**: 1203-17

34 Bandyopadhyay S and Mitra R. TargetMiner: microRNA target prediction with systematic identification of tissue-specific negative examples. *Bioinformatics* 2009; **25**: 2625-31

35 Bindea G, Mlecnik B, Hackl H, Charoentong P, Tosolini M, Kirilovsky A, et al. ClueGO: a Cytoscape plug-in to decipher functionally grouped gene ontology and pathway annotation networks. *Bioinformatics* 2009; **25**: 1091-3

36 Li J, Doyle MA, Saeed I, Wong SQ, Mar V, Goode DL, et al. Bioinformatics pipelines for targeted resequencing and whole-exome sequencing of human and mouse genomes: a virtual appliance approach for instant deployment. *PLoS One* 2014; **9**: e95217

37 Samocha KE, Robinson EB, Sanders SJ, Stevens C, Sabo A, McGrath LM, et al. A framework for the interpretation of de novo mutation in human disease. *Nat Genet* 2014; **46**: 944-50

**Supplementary Figures**

**Supplementary Figure S1.** Comparisons of DNA methylation between RA and OA. (a) A bar plot with methylation of a marker on *N4BP2*. (b) A line plot showing the density of CpGs with specific methylation levels in OA (blue) and RA (red). (c-d) Colored scatterplots comparing average methylation levels (c) and standard deviations (d) between OA and RA. Densities of individual CpGs are estimated using kernel functions and colored-scaled. (e) A heatmap with methylation profiles of the top 500 CpGs with the highest methylation variability. A dendrogram is plotted by hierarchical clustering. In the upper bar, OA is blue and RA is red. (f) A heatmap with methylation profiles of clusters classified by recursively partitioned mixture model (RPMM).

**Supplementary Figure S2.** Colored scatterplots comparing average methylation levels between WGBS data and Infinium 450K array data. Numbers in parenthesis represent Pearson correlation coefficients. Densities of individual CpGs are estimated using kernel functions and color-scaled.

**Supplementary Figure S3.** Broad inspection of large-scale DNA methylation. Using MethylSeekR Bioconductor package, partially methylated domains (PMDs), characterized by highly disordered methylation, were segmented. Each bar represents the genomic portion occupied by PMD in a sample. The disease-specific PMDs take much smaller fraction in the genome than shared PMDs. OA-specific PMDs are shown in blue and RA-specific PMDs in red.

**Supplementary Figure S4.** Subnetworks with disease-relevant phenotypes. Colors in large boxes represent gene expression levels in RA relative to OA. Red is upregulated, but green is downregulated. Stacked bar plots represent the number of DMCs or DVCs in certain genes. DMCs are colored red (hypermethylated) and blue (hypomethylated), while DVCs are colored orange (hypervariable) and green (hypovariable).

**Supplementary Figure S5.** Networks with disease-relevant miRNAs and putative target genes. Colors represent gene expression levels in RA relative to OA. Red is upregulated, but green is downregulated. Inner bar plots represent the number of DMCs or DVCs in certain genes. DMCs are colored red (hypermethylated) and blue (hypomethylated), while DVCs are colored orange (hypervariable) and green (hypovariable).

**Supplementary Tables**

**Supplementary Table S1.** Information of the patient cohort. It reports the presence of individual assays. Circles represent samples assayed, while crosses represent samples not assayed. It also contains disease status, sex, and age. A dot stands for missing information.

**Supplementary Table S2.** Summary of WGBS, miRNA-seq and exome-seq data. For WGBS, there are total number of mapped reads, CpG coverage, average CpG, methylation rate, average non-CpG methylation rate. For miRNA-seq, there is information for the number of mapped reads, percent of mapped reads located on the genome and known miRNAs. For exome-seq, there are the number of mapped reads, target coverage, mean read depth (x).

**Supplementary Table S3.** List of detected specific and shared LMRs. The genomic positions of LMRs were shown in the ‘LMR’ sheet. The cell-type specific enhancers associated with LMRs and DNA methylation changes (RA/OA) were listed in the ‘Overalp with enhancers’ sheet.

**Supplementary Table S4.** List of genes with average expression. The log2(RA/OA) values and results of statistical tests based on t-test were listed in the ‘mRNA’ sheet. For genes located near specific LMRs, it additionally contains correlation coefficients between methylation in specific LMRs and expression of genes targeted by the LMRs in the ‘Correlation’ sheet.

**Supplementary Table S5.** List of DMCs and DVCs. DMCs were CpGs with methylation difference > 0.2 and P < 2.42 x 10^-9^ (0.05/20,605,641, Bonferroni correction) in the ‘DMC’ sheet. DVCs are CpGs with FDR < 0.05 in the ‘DVC’ sheet. CpGs corresponding to both DMCs and DVCs were listed in the ‘DMC&DVC’ sheet.

**Supplementary Table S6.** List of detected DEmiRs. The log2(RA/OA) values and results of statistical tests were listed in the ‘DEmiRs’ sheet. Correlation coefficients between expression levels of DEmiRs and methylation levels in CpGs were shown in the ‘miRNA vs DNA methylation’ sheet. Expression levels of putative target genes of DEmiRs were presented in the ‘miRNA vs mRNA’ sheet.
