# Supplementary figures and images for: Epigenetic analysis in rheumatoid arthritis synoviocytes
Source: Exp Mol Med. 2019 Feb 22;51(2):22. doi: 10.1038/s12276-019-0215-5 (PMC6395697; doi:10.1038/s12276-019-0215-5)

Fig. S1 a

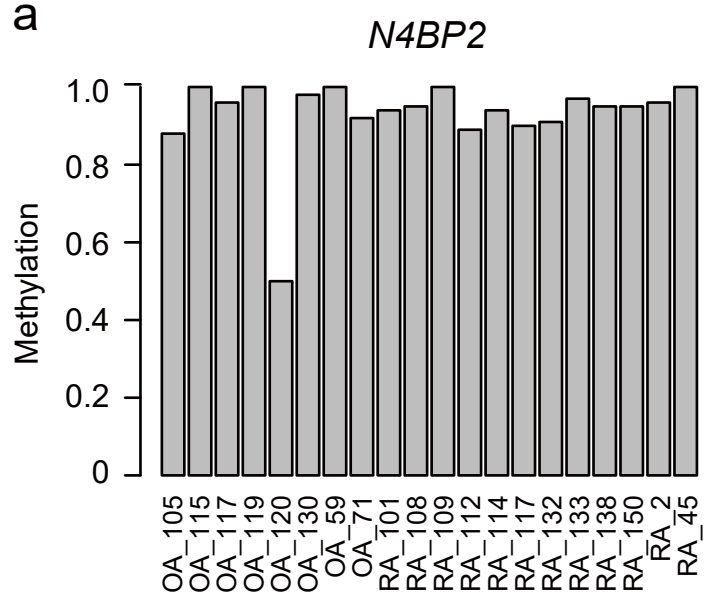

b

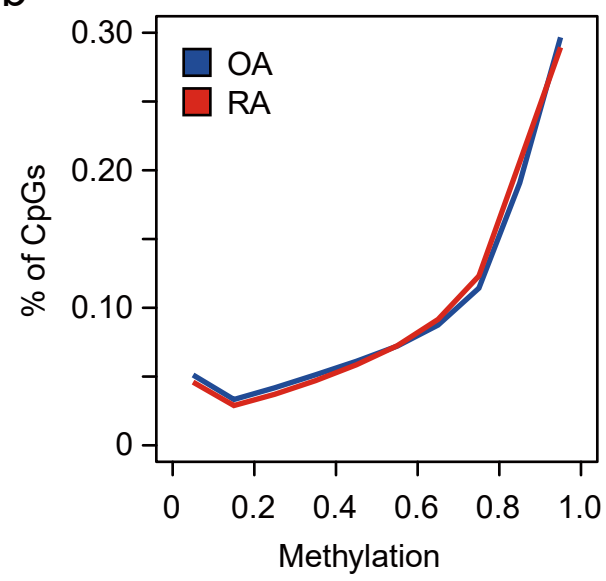

c

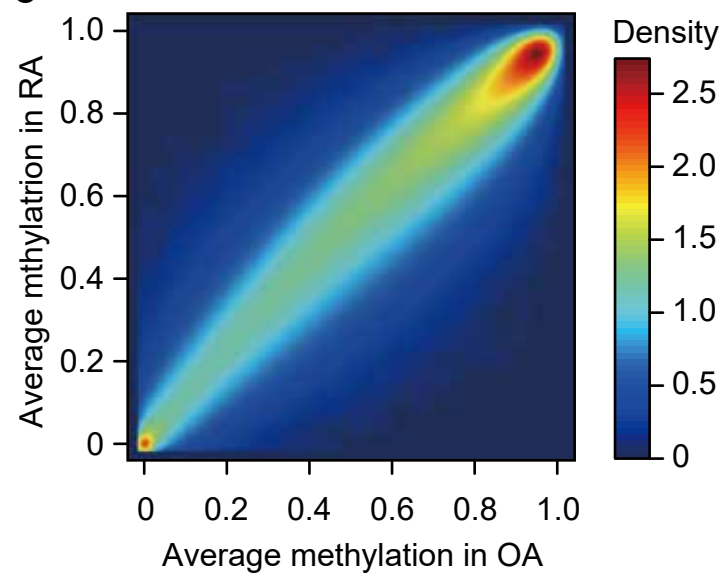

d

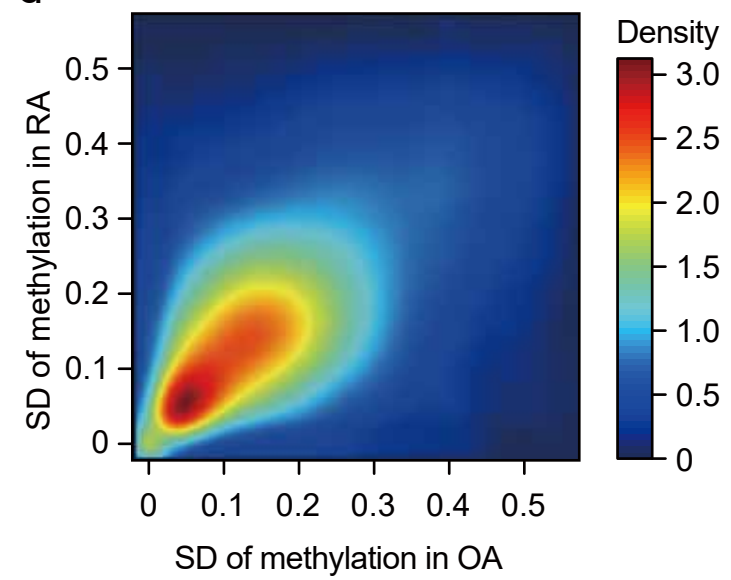

e

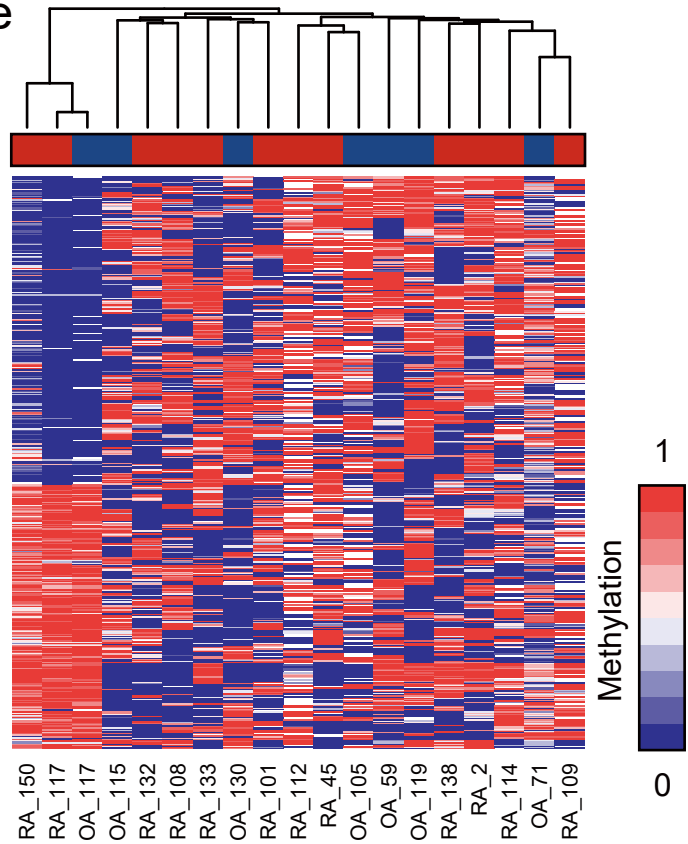

f

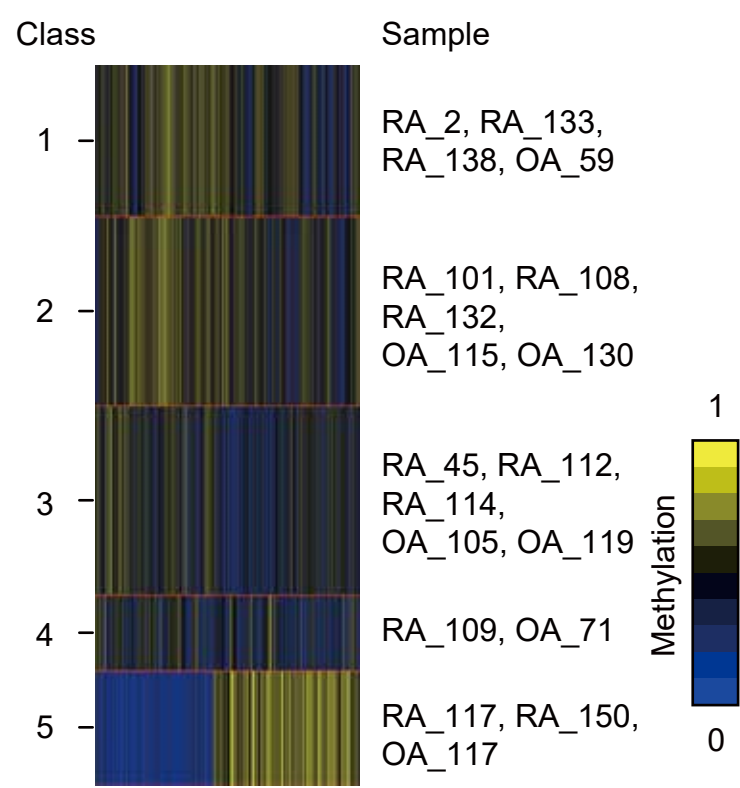

Supplement: Supplementary file 2 — Figure S1 [file 12276_2019_215_MOESM2_ESM.pdf]

Fig. S2

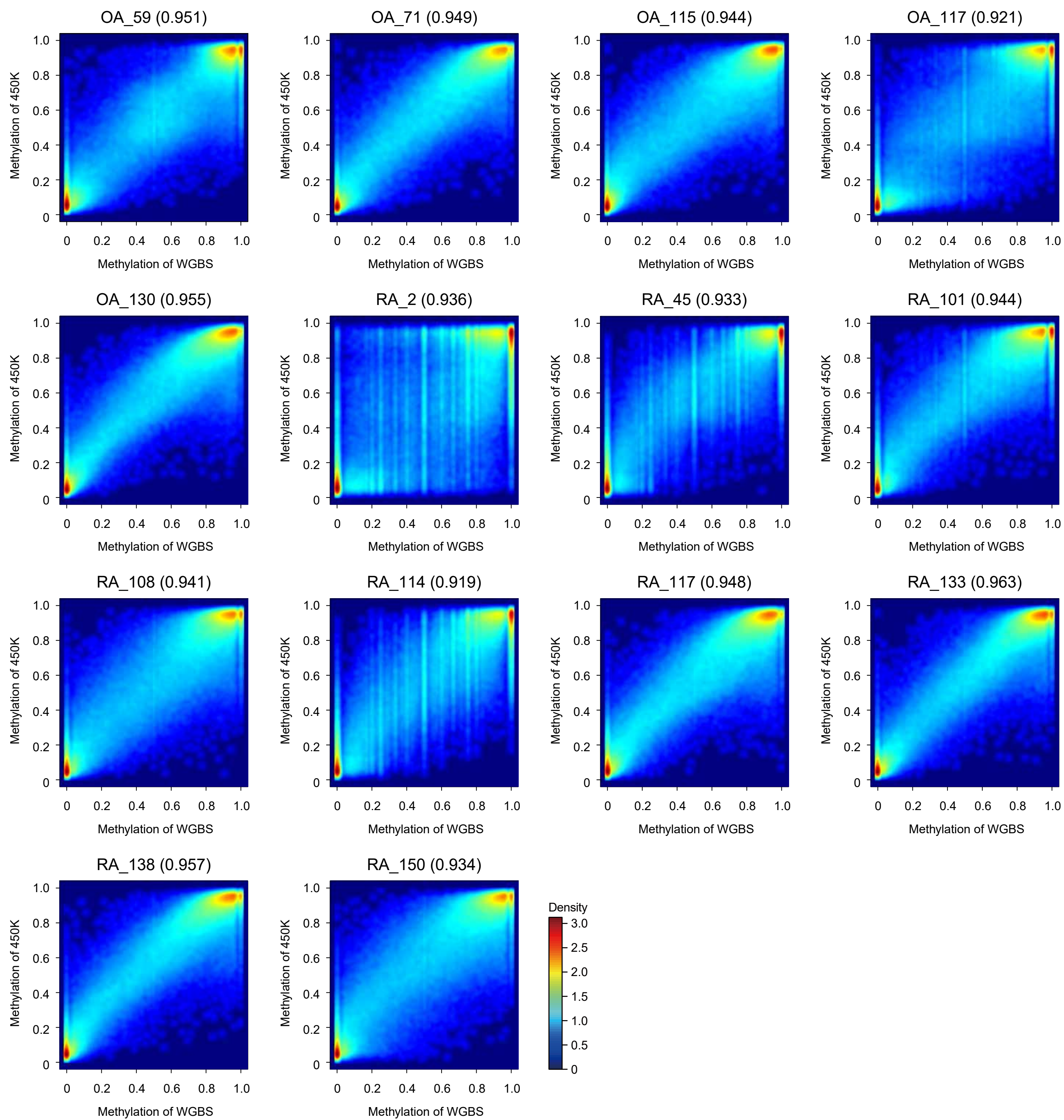

Supplement: Supplementary file 3 — Figure S2 [file 12276_2019_215_MOESM3_ESM.pdf]

Fig. S3

Disease-specific

Shared

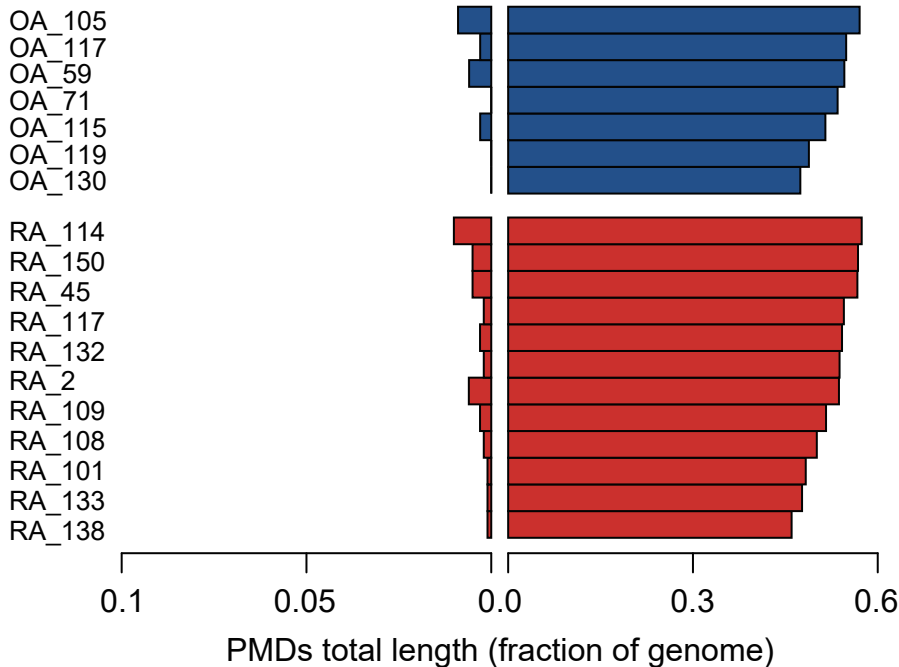

Supplement: Supplementary file 4 — Figure S3 [file 12276_2019_215_MOESM4_ESM.pdf]

Fig. S4

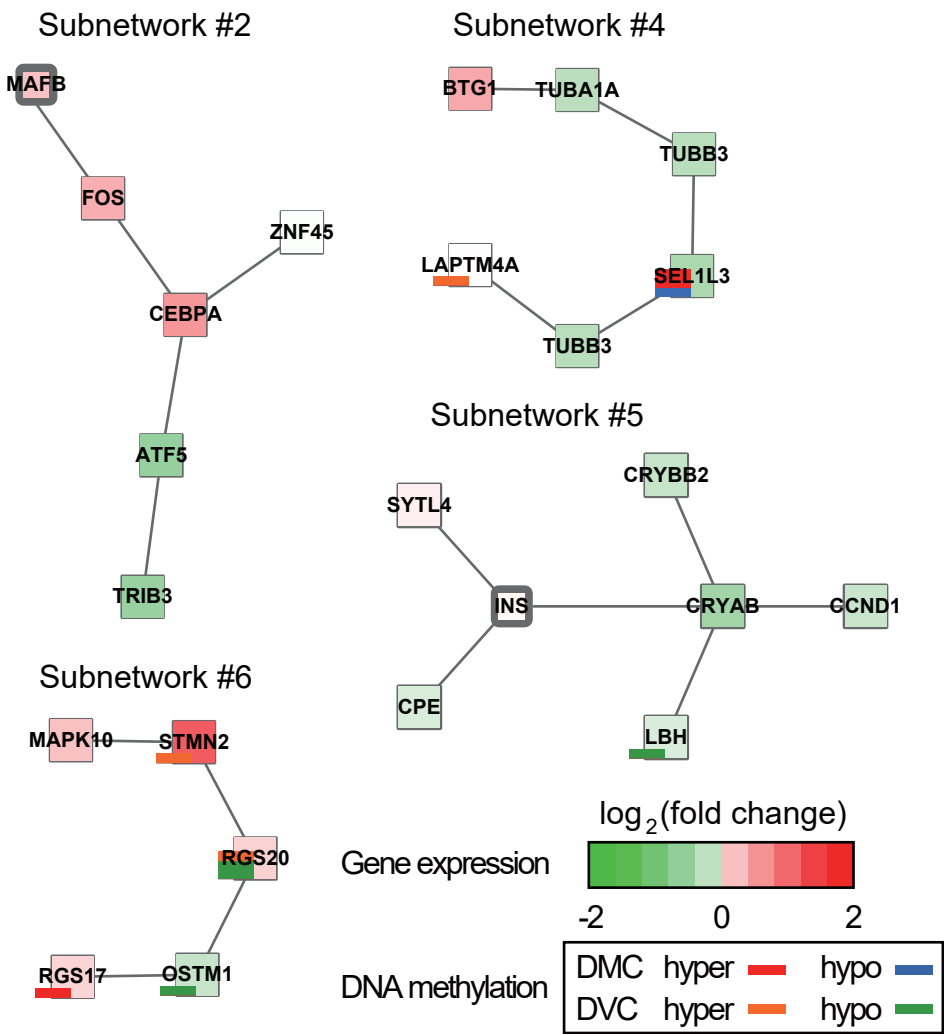

Supplement: Supplementary file 5 — Figure S4 [file 12276_2019_215_MOESM5_ESM.pdf]

Fig. S5

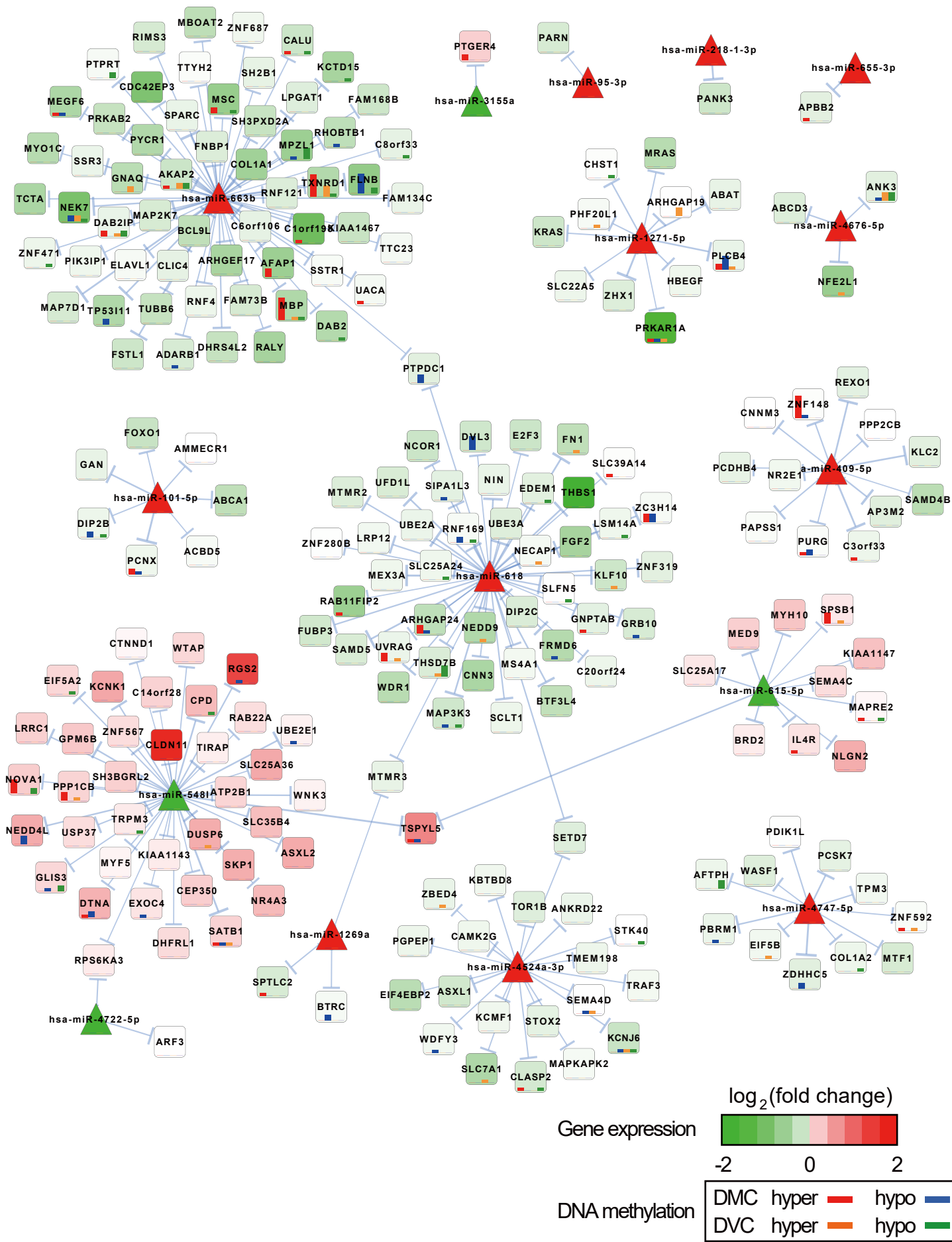

Supplement: Supplementary file 6 — Figure S5 [file 12276_2019_215_MOESM6_ESM.pdf]
